# Immune system in the intestine and mucosal inflammation

**DOI:** 10.1186/2045-7022-1-S1-S45

**Published:** 2011-08-12

**Authors:** Liam O'Mahony

**Affiliations:** 1 Swiss Institute of Allergy and Asthma Research (SIAF), University of Zurich, Molecular Immunology, Davos, Switzerland

## 

The gastrointestinal tract is home to the largest accumulation of leukocytes in the body where they are constantly being exposed to a wide array of foreign antigens. Complex signalling networks between multiple cell types ensure that the appropriate balance is maintained between immune protection from infection and tolerance of harmless antigens, such as the resident bacterial flora and dietary antigens. Disturbance of this balance results in inappropriate immune activation, as observed in patients with Inflammatory Bowel Disease or food allergy. The intestine is highly adapted to facilitate immunological sampling of intestinal contents. Specialized epithelial cells, M cells, actively transport antigen to underlying lymphoid follicles for immunological processing while dendritic cells extend dendrites between epithelial cells in order to sample adherent bacterial species. We will focus on the mechanisms by which the intestinal immune system samples luminal antigen and the controlling features that determine immunological tolerance. In particular, the molecular mechanisms by which intestinal microbes induce tolerogenic responses within the gut and the relevance of intestinal T regulatory cell responses to protection from intestinal inflammation will be discussed.

